# Failure Detection in Sensors via Variational Autoencoders and Image-Based Feature Representation

**DOI:** 10.3390/s25072175

**Published:** 2025-03-29

**Authors:** Luis Miguel Moreno Haro, Adaiton Oliveira-Filho, Bruno Agard, Antoine Tahan

**Affiliations:** 1 Laboratoire en Intelligence des Données, Department of Mathematical and Industrial Engineering, Polytechnique Montréal, Montréal, QC H3T 0A3, Canada; 2 Department of Mechanical Engineering, École de Technologie Supérieure, Montréal, QC H3C 1K3, Canada

**Keywords:** sensor failure detection, health index, variational autoencoder, feature representation, aeronautical sensors

## Abstract

This paper presents a novel approach for detecting sensor failures using image-based feature representation and the Convolutional Variational Autoencoder (CVAE) model. Existing methods are limited when analyzing multiple failure modes simultaneously or adapting to diverse sensor data. This limitation may compromise decision-making and system performance, hence the need for more flexible and resilient models. The proposed approach transforms sensor data into image-based feature representations of statistics such as mean, variance, kurtosis, entropy, skewness, and correlation. The CVAE is trained on such image representations, and the corresponding reconstruction error leads to a Health Index (HI) for detecting multiple sensor failures. Moreover, the CVAE latent space is used to define a complementary HI and a convenient visualization tool, enhancing the interpretability of the proposed approach. The evaluation of the proposed detection approach with data comprising diverse configurations of faulty sensors showed encouraging results. The proposed approach is illustrated in an industrial case study emerging from the aeronautical domain, with data from a complex electromechanical system comprising nearly 80 sensor measurements at a 1 Hz sampling rate. The results demonstrate the potential of the proposed method in detecting multiple sensor failures.

## 1. Introduction

A sensor is a specialized device that measures physical information from its environment and transforms it into analog or digital signals [1]. The resulting measurements are typically modeled as time series and may be processed and interpreted by a computing system. Sensors are essential components of data acquisition systems, allowing for applications such as condition monitoring and feedback control [2,3].

However, failures in sensors can lead to incorrect measurements, which may compromise decision-making and, ultimately, the system’s performance and integrity [4]. A sensor failure is defined as any condition in which the sensor does not properly fulfill its role or function [5]. Common types of sensor failures resulting in a partial or complete loss of functionality include bias, saturation, frozen signal, noise, and spark [6]. Possible causes of sensor failures include manufacturing defects, wear and tear from long-term use, incorrect calibration, and mishandling [7].

Detecting abnormal conditions or failures in sensors relies on accurate analyses of the measured data. The early detection of sensor failures is critical to ensure proper system operation across various engineering domains, including industrial systems [8,9], aerospace [7,10,11], energy conversion systems [12,13], and wastewater management [14], among many others. Automatic detection techniques are particularly valuable as they contribute to minimizing unexpected failures, unplanned downtime, and corresponding expenditures. Moreover, early failure detection helps mitigate their impact and implement corrective measures [2].

### 1.1. Previous Research on Sensor Failures Detection

Detecting abnormal conditions or sensor failures is a very active research avenue. Sensor failure detection models are typically categorized into three main approaches: physics-based, data-driven, and hybrid [2].

Physics-based models rely on mathematical representations of system and sensor behaviors. However, their implementation is often limited by the availability of information required to accurately describe sensor behavior, particularly in complex multivariate systems. Data-driven approaches, including statistical and Artificial Intelligence (AI)-based models, overcome these limitations by leveraging measured data [2]. Finally, hybrid approaches combine physics-based and data-driven modeling techniques to improve accuracy by leveraging complementary strengths, though they also increase model complexity.

Statistical methods such as Principal Component Analysis (PCA) effectively detect systematic errors like bias but struggle with complex, nonlinear patterns [15]. Zhao et al. [15] proposed using PCA to detect bias in sensor measurements. Normalized data are decomposed into principal components in a number determined according to their cumulative contribution. The proposed PCA implementation provided score matrices, residual matrices, and thresholds for sensor failure detection from indicators such as the Error Space Projection.

AI techniques, particularly Machine Learning (ML), have demonstrated strong potential in sensor failure detection. The availability of sensor data favors approaches based on ML models such as Artificial Neural Networks (ANNs), Support Vector Machines (SVMs), and Probabilistic Neural Networks (PNNs), to name a few. In [7], Balaban et al. use an ANN to detect several types of sensor failures, including bias, offset, and no signal. A three-layer neural network taking standardized sensor data as input is defined to distinguish the different classes of sensor failure. Zhang et al. [16] used a PNN model to detect sensor failure in railway system switches. Their algorithm synthesizes action current curves characterizing switch failures. Experimental results demonstrated the accuracy of the PNN-based switch failure detection algorithm. The authors emphasize that the proposed approach is relatively easy to implement and allows for identifying sensor failures impacting the operating current curves, which include bias, frozen measurements, and unexpected current sparks, among other irregularities [16]. In [17], Ehlenbröker et al. introduced an algorithm to evaluate the consistency between sensor values using a data fusion technique with multi-unsupervised classification levels. The proposed method combines approaches from possibility theory, fuzzification, and ML algorithms to model and detect sensor failures. The failure detection method is based on dynamic and static calculations of sensor reliability, enabling the detection of inconsistencies between sensor observations and failure types that are difficult to identify.

Autoencoders (AEs) and their variations, especially Variational Autoencoders (VAEs), have received a lot of attention in the anomaly detection literature due to their capability to compress information from high-dimensional spaces into low-dimensional latent spaces [18]. Jan et al. [19] introduced an approach using an AE to extract features from raw sensor signals. The resulting feature vector is subsequently fed to an SVM-based classification algorithm for failure identification among offset, bias, spark, and frozen sensor failures. In [20], a sensor fault detection approach based on the VAE model was introduced. The VAE is trained using sets of normal signals, learning their implicit characteristics. The reconstruction error is then used to detect faulty signals, with higher values being symptomatic of faulty conditions.

Hybrid approaches have also been explored for improved robustness. A hybrid approach for detecting sensor failures in the context of flight tests was introduced in [21]. The proposed approach combines dynamic modal decomposition and decision tree modeling. After the offline training, the resulting model is used online in real-time failure detection, covering various types of sensor failure from commercial flight data, including catastrophic failures, slow oscillations, and increased noise levels. This approach was reported to accurately detect sensor failures in commercial flight test data. Fong et al. [22] presented a hybrid approach for detecting and diagnosing sensor failures in cooling installations with a hybrid approach combining ML and statistical methods. The proposed method is based on the fusion of data from multiple sensors, including temperature, pressure, and flow measurements within a cooling plant. It allows the detection of sensor failures impacting critical components. Chen et al. [23] used an SVM classifier to categorize features from multiple aircraft engine sensors. The model was trained with a labeled database comprising 500 signals, including healthy-condition data and sensor failure modes such as bias and spark. Reddy et al. [24] introduced a threshold-based approach to specifically detect sensor saturation. The detection algorithm is combined with the classification of saturated segments according to their duration (short-, medium-, or long-duration saturation), which helps assess the impact of saturated sensor data on the overall analysis.

Besides sensor failure detection, some works also address the replacement, adaptation, or correction of incorrect sensor information [25,26,27]. Feng et al. introduced a sensor failure detection and reconciliation algorithm to analyze data from clinical experiments with artificial pancreas systems in individuals with diabetes. The proposed algorithm uses ANNs to analyze residuals and classify diverse sensor conditions. It integrates four modeling techniques: a robust Kalman filter for outliers, locally weighted partial least squares, a subspace-based predictor method, and kernel-based recursive least squares with an approximate linear dependence criterion. The authors evaluated the approach on clinical data comprising 896 h of continuous glucose monitoring from multiple experiments, demonstrating that in addition to detecting and diagnosing sensor failures, the method can more accurately reconcile erroneous sensor signals with model-estimated values [26]. In [28], the authors divided the sensors into two groups: a set containing sensors susceptible to failure and a set of reliable sensors. The proposed approach was based on Multi-Layer Perceptron Neural Networks. It was trained and validated with real-world data, focusing on sensor bias. Their approach allows for the detection, isolation, and adaptation of sensor failures and comprises three main steps: (i) creation of virtual sensors to estimate the measurements of unreliable sensors and, if applicable, exclusion of the respective measurements, (ii) the calculation of residuals indicating the dissimilarity between the virtual sensors estimates and the actual measurements, and (iii) the classification and decision-making based on residual analysis to detect and isolate sensor failures [28].

### 1.2. Paper Motivation and Outline

Despite significant advancements in sensor failure detection, some limitations remain to be addressed. Many approaches are designed to detect specific types of failures, limiting their applicability in real-world industrial systems where multiple failure modes can co-occur [29]. Statistical techniques like PCA and Kalman filtering struggle with complex, high-dimensional failure scenarios, while ML models require large labeled datasets and lack interpretability. Given the increasing complexity of sensor networks, there is a clear need for a more flexible and robust failure detection approach.

This research addressed these challenges by proposing a novel sensor failure detection methodology, leveraging an image-based feature representation and the Convolutional Variational Autoencoder (CVAE) model. The key contributions of this work are outlined as follows:

1. Failure type: The proposed approach unifies the detection of multiple types of sensor failure, including no signal, bias, frozen, noise, spark, and saturation.
2. Feature representation: Statistical features—mean, variance, entropy, skewness, kurtosis, and correlation—are extracted from sensor time series and converted into pixel matrices. This approach enables the use of convolutional neural network layers for effective failure detection and captures complex relationships in sensor data.
3. CVAE model for failure detection: The CVAE model was designed to detect failures in sensor data. The CVAE model is defined and trained to learn data distribution from sensors in normal condition, identifying failures based on reconstruction errors. This definition enables a robust and adaptable failure detection framework.
4. Validation: The proposed method is evaluated using synthetic failure data and a real-world industrial dataset from a complex electromechanical system from the aeronautical domain.

This paper is organized as follows: Section 2 describes types of sensor failures and key features for vibration analysis. Section 3 presents the proposed detection approach. Section 4 presents the results and discussion and is followed by the conclusion in Section 5.

## 2. Background: Classification of Sensor Failures

**Figure 1:**
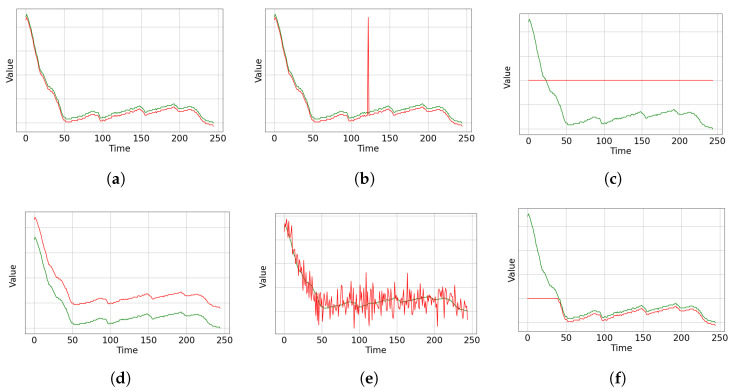
Measured time series (red) and actual physical phenomena (green) for multiple sensor conditions: (**a**) sensor in healthy condition; (**b**) sensor with spark failure; (**c**) sensor with frozen failure; (**d**) sensor with bias failure; (**e**) sensor with noise failure; (**f**) sensor with saturation failure.

A sensor failure results in unexpected measurements of the observed signal output, even when the system under analysis operates under normal conditions. Various types of sensor failures can occur. Figure 1 illustrates a signal from a properly functioning sensor alongside signals exhibiting different failure modes.

Let r(t) be the current signal of an (unknown) physical phenomenon. The signal measured by a sensor at instant *t*, m(t), is given by

(1) m(t)=r(t)+ϵ,

where ϵ is the measurement noise at *t* [7].

In an ideal scenario, the actual signal r(t) is exactly the measured signal m(t), thus ϵ=0. In practice, however, the sensor is considered operating in a healthy state when ϵ is small compared to r(t), i.e., $|\epsilon /\max_{t}\left(r\left(t\right)\right)|\ll 1$. Figure 1a illustrates this condition. Sensor failures can be categorized according to the pattern of measurements over time as spark, frozen, bias, noise, or saturation. A no-signal or communication loss failure occurs when no signal is available.

### 2.1. Spark

The spark failure in a sensor signal (see Figure 1b) can be modeled as a sudden, brief disturbance in the sensor measurement [30]. This disturbance is represented mathematically using the impulse function δ(t) [31]. The signal m(t) is then given by

(2) $$m\left(t\right)=r\left(t\right)+A\cdot \delta \left(t-t_{0}\right).$$

where *A* is the spark pulse amplitude and $t_{0}$ the instant when it occurs.

### 2.2. Frozen

Frozen sensor refers to a type of failure when the data series measured by the sensor is constant over a range of time, as depicted in Figure 1c [32]. The general equation is given by

(3) $$m\left(t\right)=\left\{\begin{array}{cc} r(t), & \mathrm{if}\;t<t_{1}\\ C, & \mathrm{if}\;t_{1}\leq t\leq t_{2}\\ r(t), & t>t_{2}, \end{array}\right.$$

where C∈R represents the constant value measured during the interval $\left[t_{1},t_{2}\right]$.

### 2.3. Bias

A biased sensor is such that its measure is shifted by a constant offset (systematic error) [7], as depicted in Figure 1d and given by

(4) $$m\left(t\right)=r\left(t\right)+\theta_{0},$$

where $\theta_{0}\in R$ is a constant value.

### 2.4. Excessive Noise

Figure 1e depicts an excessively noisy sensor measurement. The environment in which sensors operate can be complex and sensitive to various sources of noise, including internal noise, hardware noise, and ambient noise. Internal noise mainly comes from sensor and circuit component characteristics, such as noise generated by amplifiers. External noise, on the other hand, comes from human or environmental interference outside the sensor circuit. Although noise is common in sensor data, an abnormally high noise level can cause problems in sensor signals and limit the performance obtained from a given device [33]. Given the measurement noise $\epsilon^{*}$, with $|\epsilon^{*}/\max_{t}\left(r\left(t\right)\right)|\gg 1$, the excessive noise sensor failure can be modeled by

(5) $$m\left(t\right)=r\left(t\right)+\epsilon^{*}$$

### 2.5. Saturation

Saturation (see Figure 1f) occurs when a sensor is exposed to values beyond its measurement range [34]. In such cases, the sensor output remains fixed at either its maximum or minimum limit, as given by

(6) $$m\left(t\right)=\left\{\begin{array}{cc} \mathrm{Max}\;\mathrm{value}, & \mathrm{if}\;r(t)>\mathrm{Max}\;\mathrm{value}\\ r(t), & \mathrm{if}\;\mathrm{Min}\;\mathrm{value}\leq r(t)\leq \mathrm{Max}\;\mathrm{value}\\ \mathrm{Min}\;\mathrm{value}, & \mathrm{if}\;r(t)<\mathrm{Min}\;\mathrm{value}, \end{array}\right.$$

where “Max value” and “Min value” are the upper and lower limits of the sensor measurements [34]. In some cases, saturation can occur if the power or intensity of the signal reaching the sensor is too high [35].

Real-world occurrences of sensor failures are presented below to illustrate the different failure types:

- Spark failure: A sudden spike in sensor readings due to electromagnetic interference or transient power surges.
- Frozen failure: The sensor output remains constant despite actual variations, often caused by communication loss or hardware malfunction.
- Bias failure: A consistent deviation from the actual value, typically resulting from calibration errors.
- Noise failure: Random fluctuations in sensor readings due to external disturbances or aging components.
- Saturation failure: The sensor reaches its upper or lower limit and remains at that value, failing to capture further variations.

## 3. Proposed Approach for Detecting Sensor Failures

This study introduces an approach for detecting sensor failures. A sensor is considered normal or healthy when its measurements follow expected operational behavior, showing no deviations from the historical patterns observed in healthy systems. In contrast, a sensor is classified as abnormal when it exhibits deviations compared to a healthy reference signal. These anomalies can manifest as unexpected variations in statistical features.

**Figure 2:**
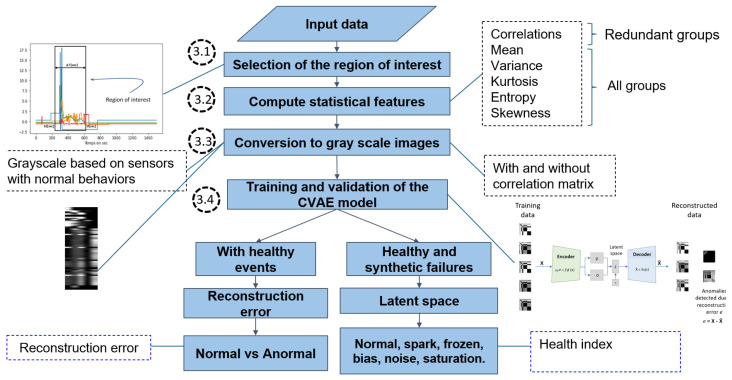
Flowchart of the proposed approach for detecting sensor failures in industrial systems.

The proposed detection approach consists of two phases: online and offline. The online phase pre-processes the data and trains the CVAE model. The offline phase performs failure detection. Figure 2 depicts the flowchart of the proposed approach. The four steps of the online phase are described in Section 3.1, Section 3.2, Section 3.3 and Section 3.4. The offline detection phase consists of two branches corresponding to complementary HIs using the reconstruction error and the CVAE latent space.

This study analyzes discrete time series corresponding to measurements acquired by multiple sensors under diverse operational conditions. An underlying hypothesis of the proposed approach is that the different events share certain similarities, which justifies examining the data from various events comparatively to characterize their respective patterns. The aim of learning from measurements across multiple events is to detect abnormal events with signatures in specific sensors. The proposed approach is suitable for multiple industrial systems, including engines, industrial production lines, and mechanisms.

### 3.1. Selection of the Time Window of Interest

The selection of the time window of interest is a crucial initial step in analyzing time series data associated with specific events. This process involves identifying the pertinent time period for each event, effectively isolating a time window that captures relevant information.

The event boundaries are defined by analyzing the sensor data. This step requires understanding the specific characteristics of the event and determining when significant measurements begin and cease. The corresponding time window is isolated once the start and end points are established. This procedure ensures that irrelevant data outside this window do not influence the results. Furthermore, the time window length can be tailored to specific applications by adjusting pre-event and post-event durations.

### 3.2. Compute Features

This study focuses on events of interest and their occurrences within the dataset under analysis. Specific sensor measurements characterize each event. Statistical features provide a compact and informative representation of time series data, capturing essential signal characteristics such as distribution, variability, and shape. They help summarize large amounts of data efficiently, making it easier to detect deviations that indicate sensor failures [36]. Following the state-of-the-art vibratory analysis, five statistical measures were chosen to describe the information from the time series: mean, variance, kurtosis, entropy, and skewness. Moreover, the correlation between these sensors is also used to tackle eventual redundancy between sensors.

Let $x_{ij}\left(t_{k}\right)$ represent the measurement from sensor *i* for event *j* at time $t_{k}$, where $i\in \{1,\ldots ,N_{c}\}$, $j\in \{1,\ldots ,N_{v}\}$, and $k\in \{1,\ldots ,N_{d}\}$. The statistical features extracted from the time series are listed below:

- Mean value ${\bar{x}}_{ij}$:
  (7) $${\bar{x}}_{ij}=\frac{1}{N_{d}}\sum_{k=1}^{N_{d}}x_{ij}\left(t_{k}\right)$$
- Variance $\mathrm{VAR}(x_{ij})$:
  (8) $$\mathrm{VAR}\left(x_{ij}\right)=\frac{1}{N_{d}-1}\sum_{k=1}^{N_{d}}{\left(x_{ij}\left(t_{k}\right)-{\bar{x}}_{ij}\right)}^{2}$$
- Skewness $S(x_{ij})$:
  (9) $$S\left(x_{ij}\right)=\frac{\frac{1}{N_{d}-1}\sum_{k=1}^{N_{d}}{\left(x_{ij}\left(t_{k}\right)-{\bar{x}}_{ij}\right)}^{3}}{{\left(\frac{1}{N_{d}-1}\sum_{k=1}^{N_{d}}{\left(x_{ij}\left(t_{k}\right)-{\bar{x}}_{ij}\right)}^{2}\right)}^{\frac{3}{2}}}$$
- Kurtosis $K(x_{ij})$:
  (10) $$K\left(x_{ij}\right)=\frac{1}{N_{d}-1}\sum_{k=1}^{N_{d}}{\left(\frac{x_{ij}\left(t_{k}\right)-{\bar{x}}_{ij}}{\sqrt{VAR(x_{ij})}}\right)}^{4}$$
- Entropy $H(x_{ij})$, where $p(x_{ij})$ is the empirical probability density:
  (11) $$H\left(x_{ij}\right)=-\sum_{k=1}^{N_{d}}p\left(x_{ij}\left(t_{k}\right)\right).\log\left(p\left(x_{ij}\left(t_{k}\right)\right)\right)$$
- Pearson correlation $CORR(x_{pj},x_{qj})$ for an event *j* given a pair of sensor measurement time series $X_{pj}$ and $X_{qj}$:
  (12) $$CORR\left(x_{pj},x_{qj}\right)=\frac{\sum_{k=1}^{N_{d}}\left(x_{qj}\left(t_{k}\right)-{\bar{x}}_{qj}\right)\left(x_{pj}\left(t_{k}\right)-{\bar{x}}_{pj}\right)}{\sqrt{\sum_{k=1}^{N_{d}}{\left(x_{qj}\left(t_{k}\right)-{\bar{x}}_{qj}\right)}^{2}\sum_{k=1}^{N_{d}}{\left(x_{pj}\left(t_{k}\right)-{\bar{x}}_{pj}\right)}^{2}}}$$

The retained features quantify variations in signal distribution, randomness, and deviation from normal behavior, making them well suited for detecting anomalies and distinguishing various types of failure. For instance, mean and variance help identify gradual shifts and saturation effects, while kurtosis and entropy highlight sudden fluctuations and irregularities. Correlation measures deviations from expected patterns, reinforcing the detection of abnormal trends. Moreover, compared to dynamic features requiring complex time series modeling, the statistical descriptors provide a straightforward and interpretable representation of the information from the acquired data.

### 3.3. Grayscale Image Representation of the Features

The creation of a grayscale image representation for each event involves two key steps: normalization and pixel value mapping. Normalization is performed across all events to ensure consistency in the descriptor scale, allowing meaningful comparisons between events [37]. Let $X_{ij}={\left[x_{ij}\left(t_{1}\right),x_{ij}\left(t_{2}\right),\ldots ,x_{ij}\left(t_{D}\right)\right]}^{T}$ be the matrix gathering measurements for all sensors $i\in \{1,\ldots ,N_{c}\}$ and events $j\in \{1,\ldots ,N_{v}\}$; the normalization of the measurement $x_{ij}$ is given by $y_{ij}$:

(13) $$y_{ij}=\frac{x_{ij}-\min\left(Xij\right)}{\max\left(Xij\right)-\min\left(X_{ij}\right)}$$

The pixel value mapping consists of multiplying the normalized values to convert them into pixel intensities, where black corresponds to zero (the minimum value) and white corresponds to the maximum value. Furthermore, the pixel is set to 0 (black) when the minimum and maximum values are equal [37]. The pixel value $P_{ij}$ corresponding to the measurement $x_{ij}$ is given by

(14) $$P_{ij}=255\;\frac{x_{ij}-\min\left(Xij\right)}{\max\left(Xij\right)-\min\left(X_{ij}\right)}=255\;y_{ij}$$

**Figure 3:**
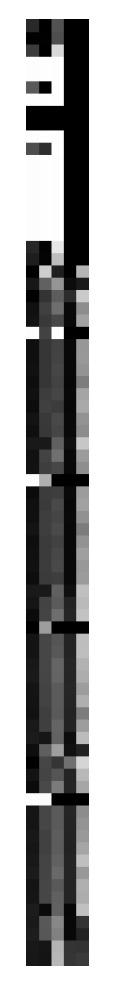
Grayscale image representation of the operational condition considering 77 sensors and 5 features. The (i,j) pixel represents the statistic feature *j* estimated for the sensor *i*.

Figure 3 depicts a grayscale image of feature descriptors from a complex electromechanical system equipped with 77 sensors. This image has a pixel size of 5×77, corresponding to five features and 77 sensors monitoring different operational parameters.

This structured representation is part of the preparation of the model input and was designed to provide a more comprehensive view of the data, therefore facilitating pattern recognition. The normalization ensures that the pixel values represent relative differences across all events, not just within a single event, while the intensity of pixels reflects the magnitude of the descriptor.

### 3.4. Training and Validation of the CVAE Model

Image representations generated from the statistical features leverage the ability of CNNs to learn complex spatial representations. Although the original data are not conventional images, converting them into pixel arrays enables CNNs to extract spatial features and identify patterns. This transformation also enables advanced image-processing techniques. Similarly, representing time series data as images allows the CVAE model to capture underlying structures, enhancing the detection of failures, unexpected transitions, or sensor behavior changes over time.

**Figure 4:**
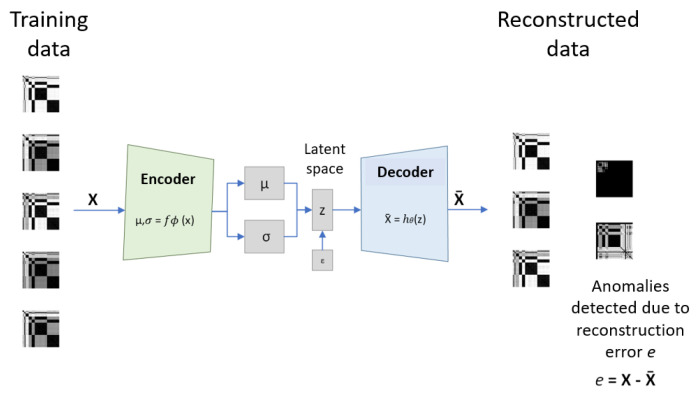
Training and validation process using the CVAE reconstruction error.

The CVAE consists of two main components: the encoder and the decoder. The encoder processes the input images and compresses them into a latent space representation, capturing the essential features of the data. The decoder then reconstructs the images from this latent space. The CVAE training forces it to capture meaningful information from the input images. Figure 4 provides a schematic representation of the CVAE structure and uses.

The encoder is a neural network with parameters ϕ that maps the elements of $X\in R^{n_{F}}$ into the latent space with average $\mu \in R^{n_{L}}$ and standard deviation $\sigma \in R^{n_{L}}$:

(15) $$f_{\phi }:X\mapsto \left\{\mu ,\sigma \right\},R^{n_{F}}\to R^{n_{L}}\times R^{n_{L}}$$

where $n_{F}$ is the dimension of the input data and $n_{L}$ is the dimension of the latent space, with $n_{L}<n_{F}$. The reparameterization trick introduces the variational Bayesian component into the latent space. It maps μ, σ, and the Gaussian vector of dimension $n_{L}$ϵ∼N(0,1) into the latent variable $z\in R^{n_{L}}$:

(16) $$g:\left\{\mu ,\sigma ,\epsilon \right\}\mapsto z=\mu +\sigma ⊙\epsilon ,R^{n_{L}}\times R^{n_{L}}\times E\to R^{n_{L}}.$$

where ϵ∼N(0,1) is defined on the sample space E and ⊙ is the elementwise product. Finally, the CVAE decoder $h_{\theta }$ is a Deep Neural Network (DNN) with parameters θ mapping z into the CVAE output $\bar{X}$:

(17) $$h_{\theta }:z\mapsto \bar{X},R^{n_{L}}\to R^{n_{F}}.$$

The process of training and validation of the CVAE model is as follows:

1. Data preparation: Transformation of the sensor time series data into images based on statistical features. Data are partitioned into training, validation, and test datasets.
2. Model architecture: An initial architecture is defined based on input data dimension, choices for the latent space dimension, and guidelines from previous implementations.
3. Training the model: Given a suitable loss function, optimization of model parameters using the Adam algorithm. Model weights are updated based on the computed loss through the training epochs. The CVAE loss function quantifies how well the model reconstructs the input images (reconstruction error) and how closely the latent distribution aligns with a prior distribution (Kullback–Leibler error).
4. Evaluation: The evaluation process involves estimating the model’s performance on the test dataset, which was not seen during the training process. Based on the observed performances, the model architecture and training hyperparameters may be adjusted before a new training iteration.

The CVAE enables the robust detection of sensor failures through the analysis of the reconstruction error and the latent space manifold [38]. Additionally, its decoder can be used for sample generation by synthesizing new data points from coordinates in the latent space. The latent space projection also eases the categorization of the system condition, allowing the implementation of classification models when labeled data are available.

### 3.5. Detection of Sensor Failures

Models such as the AE, the VAE, and their variations allow for detecting abnormal conditions through the reconstruction error estimated on new data [39]. The VAE incorporates a regularization of the latent space distribution, allowing for using the latent space itself as an analysis tool [40]. The present work combines a reconstruction-error-based detection and a CVAE-latent-space-based detection and classification of sensor failure.

#### 3.5.1. Reconstruction Error Method

The model trained on healthy-condition data learns the relations characterizing the corresponding behavior. The reconstruction error $L_{RE}=X-\bar{X}$ can then be used to evaluate how well the model reconstructs new data [41]. High $L_{RE}$ indicates abnormal inputs and can be associated with faulty sensors. Indeed, the model struggles to accurately reconstruct new data when it is unusual compared to normal instances in the dataset. A threshold is set for the $L_{RE}$ value to detect sensor failures. Data producing a reconstruction error higher than this threshold are considered faulty.

#### 3.5.2. Latent Space-Based Detection

**Figure 5:**
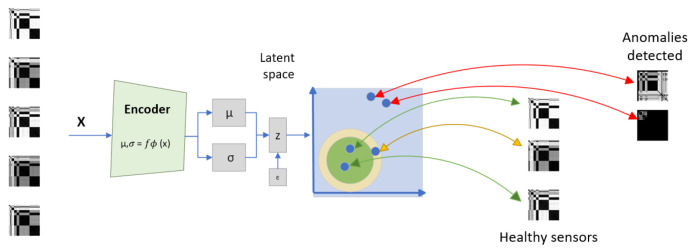
CVAE latent space projection. Healthy sensor data cluster centrally (green), incipient faults appear at the frontier (yellow), and anomalies are mapped further away (red). Colored arrows indicate the mapping of samples.

The CVAE latent space provides a low-dimensional representation of the input data, capturing the most relevant information from the original high-dimensional data, as illustrated in Figure 5.

The CVAE latent space is such that input images sharing similar characteristics are encoded close together. Therefore, input images from similar conditions are mapped into specific clusters in the latent space. Moreover, when the CVAE is trained to represent normal and multiple abnormal conditions, it often projects the normal-condition cluster in a central position, and data from the other conditions are mapped into clusters surrounding the healthy-condition cluster [42].

The CVAE’s ability to learn latent representations makes it useful for anomaly detection. During the training phase, the CVAE learns to project images from multiple conditions in the latent space, providing a meaningful representation of the system’s health condition [42]. A Health Index (HI) can be defined using the Euclidean norm of the data points in a standardized latent space, such as the standard Nataf space [43]. It enables the detection of deviations from the normal condition, indicating potential degradations or failures. Different types of HIs can be used to identify rapid or gradual changes in the system’s behavior, depending on the system under analysis [43].

In this work, the $\chi^{2}$ statistical test is used to calculate the HI threshold, with a degree of freedom equal to the CVAE latent space dimension. Confidence levels of 90% and 95% were chosen to determine the thresholds for anomaly detection. These significance levels suit quantifying the confidence with which an event can be classified as abnormal while balancing false-positive and false-negative rates. By setting these thresholds, one can ensure the decision boundary for classification is statistically sound and not due to random variations in the data.

The $\chi^{2}$ test assesses whether observed frequencies significantly differ from expected frequencies under a defined theoretical distribution. For a CVAE projecting datasets from multiple conditions, the distribution of all points projected in the latent space approximately follows a multivariate normal distribution. Expected frequencies are derived from the latent space projection of the healthy dataset, while observed frequencies are estimated from newly observed datasets under analysis. The $\chi^{2}$ statistic is computed as given by

(18) $$\chi^{2}=\sum \frac{{\left(O_{i}-E_{i}\right)}^{2}}{E_{i}}$$

where $O_{i}$ represents the frequencies observed in each category, and $E_{i}$ denotes the frequencies expected based on the theoretical distribution. This statistic evaluates the null hypothesis, determining if deviations are statistically significant, thus ensuring the robustness and reliability of the anomaly detection thresholds.

## 4. Results and Discussion

This study investigated two configurations of the input data: one that considers sensor correlation and one that excludes it. In the first configuration, statistical features—mean, variance, kurtosis, entropy, and skewness—are computed for each sensor’s time series. In addition to these, the second configuration incorporates inter-sensor correlations. Both input configurations were used to generate the image representation of the statistical features, train the CVAE model, and implement the proposed detection and visualization approach.

A real-life case study from the aeronautical domain was used to evaluate the proposed approach. The proprietary dataset corresponds to physical measurements from an electromechanical aeronautical system. This dataset consists of time series from 77 sensors of various types, with a sampling frequency of 1 Hz and an acquisition period of 25 min. Of over 8000 recorded events, only 12 were reported as abnormal. The imbalance of this database, with a predominance of healthy-condition datasets, reflects one of the main challenges in real-life applications. Moreover, synthetic sensor failures were created for bias, spark, frozen, noise, and saturation sensor failures, as defined in Section 2.

### 4.1. Feature-Based Images from Sensors Data

**Figure 6:**
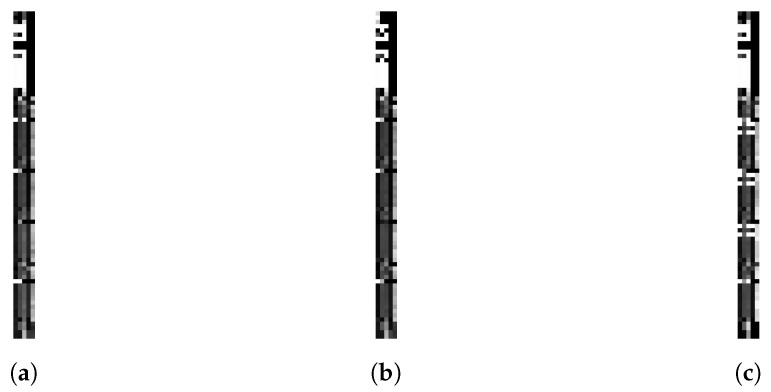
Grayscale image representation considering 77 sensors and 5 features for different sensor conditions: (**a**) healthy, (**b**) frozen, and (**c**) spark.

Figure 6 depicts the image representation of the statistical features generated from the real-life case study dataset.

The intricate patterns in these figures evidence the difficulty of analyzing the system conditions directly from the retained descriptors. Different patterns in these image representations indicate changes in the sensor condition, therefore suiting the definition of sensor failure detection approaches [44].

### 4.2. CVAE Architecture and Training

The implementation of the CVAE model followed guidelines from [45,46]. Accordingly, the CVAE architecture and training hyperparameters were selected to ensure model convergence and generalization on the training and validation datasets.

The CVAE input dimension for the five-feature image representation is 5×77. The latent space dimension was set to latent_dim=3 to enable direct plotting. The CVAE encoder consists of five Conv2D layers set with progressively increasing filter sizes and strides for downsampling, followed by a flattening layer to transform the output into a 1D vector. This resulting vector is then fed into a 256-node Dense layer. Each Conv2D layer employs the Rectified Linear Unit (ReLU) activation function. A Dropout layer with a 0.5 dropout rate was incorporated to prevent overfitting. The decoder reconstructs the original input from the latent space representation. Its input layer with dimension latent_dim is followed by a 128×5×77 Dense layer set with the ReLU activation function. This layer is reshaped into a 3D tensor of dimensions 5×77×128 to facilitate the subsequent Conv2DTranspose layers. The decoder mirrors the encoder in structure, employing Conv2DTranspose layers with decreasing filter sizes and strides to upsample the representation back to the original image dimension of 5×77. The final Conv2DTranspose layer outputs the reconstructed image using a sigmoid activation function. The CVAE training was set with 20 epochs and a batch size of 32.

The configurations for the six-feature image representations are identical except for the feature-related input and output dimension (six instead of five).

### 4.3. Reconstruction Error

**Figure 7:**
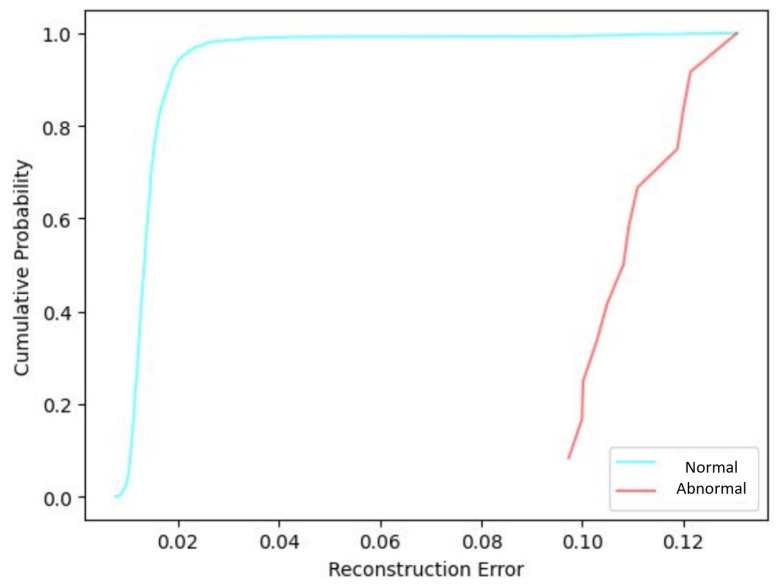
Reconstruction error CDF for the CVAE model using 5-feature input images.

Following the CVAE model training, the reconstruction errors were analyzed based on their Cumulative Distribution Function (CDF). Figure 7 presents the reconstruction error CDF corresponding to the five-feature image representation for sensors in normal and abnormal conditions.

The normal event exhibits a consistently lower reconstruction error, with the CDF reaching nearly 1.0 at a relatively small error value (around 0.025). This distribution contrasts with the abnormal sensor reconstruction error CDF. The separation between the two curves demonstrates the effectiveness of the CVAE in identifying failures based on reconstruction error, with abnormal events deviating substantially from the model’s learned distribution.

**Figure 8:**
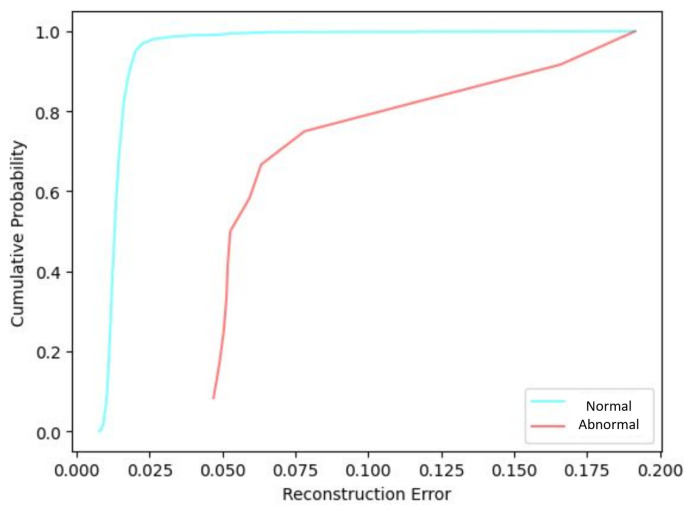
Reconstruction error CDF for the CVAE model using six-feature input images.

Figure 8 presents the CDF of the reconstruction error corresponding to the six-feature image representation for normal and abnormal sensors.

The pattern of normal-condition reconstruction error CDF is similar for five-feature and six-feature image representations, with the normal-condition sensors showing consistently lower reconstruction error levels than the abnormal conditions. The comparison between the reconstruction error CDF for five- and six-feature images shows that the distribution is shifted toward smaller error values for the six-feature model.

### 4.4. CVAE Latent Space Projection and Distance Metric

**Figure 9:**
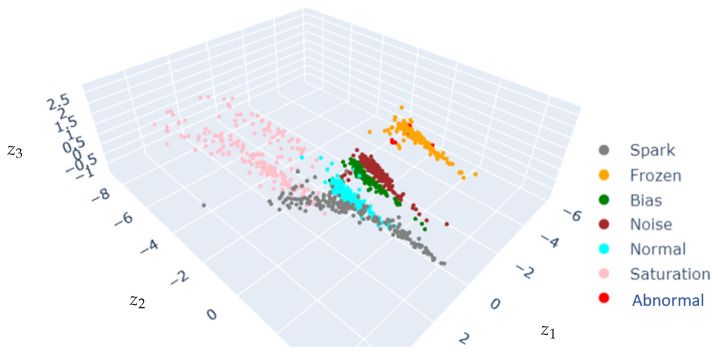
Latent space projection using 5-feature images.

Figure 9 shows the CVAE latent space projecting the database. Different sensor conditions are projected into disentangled clusters in the latent space, and clusters corresponding to healthy sensors are surrounded by sensor failure clusters.

**Figure 10:**
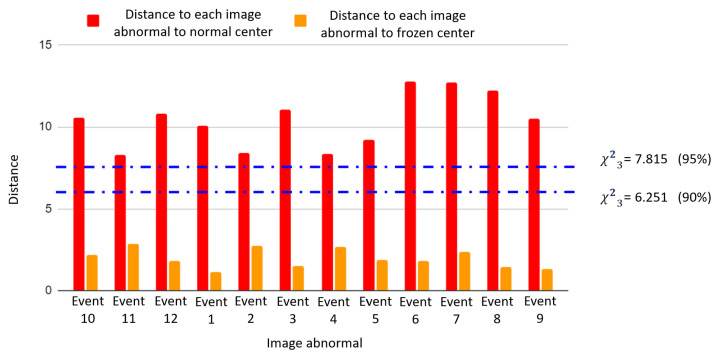
Based on the 5-feature image representation, distances from projected events to the center of the healthy condition (red) and the center of the frozen sensor fault (orange).

The HI is defined as the distance between each image labeled as abnormal and the group’s center labeled as normal. The results are shown in Figure 10. The blue dashed lines indicate the significance levels 95% and 90% for the $\chi^{2}$ test with 3 degrees of freedom.

Figure 10 shows that the distance of each of the images labeled as abnormal to the center of the normal-condition cluster is greater than the threshold set from the HI distribution on normal-condition data. In addition, the distance of each abnormal image to the center of the frozen sensor cluster is smaller than the threshold.

**Figure 11:**
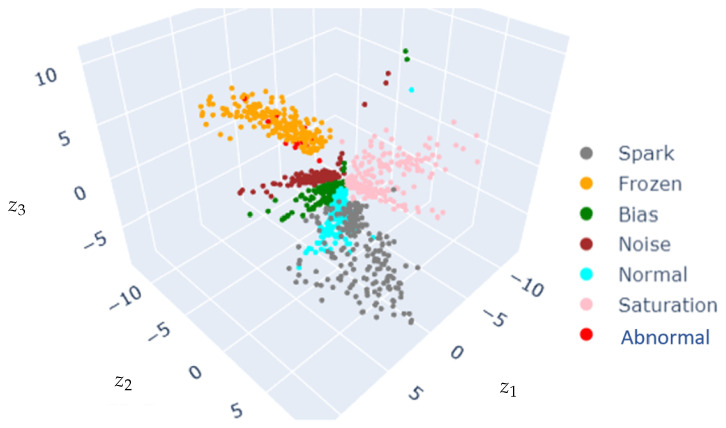
Latent space projection using 6-feature images.

**Figure 12:**
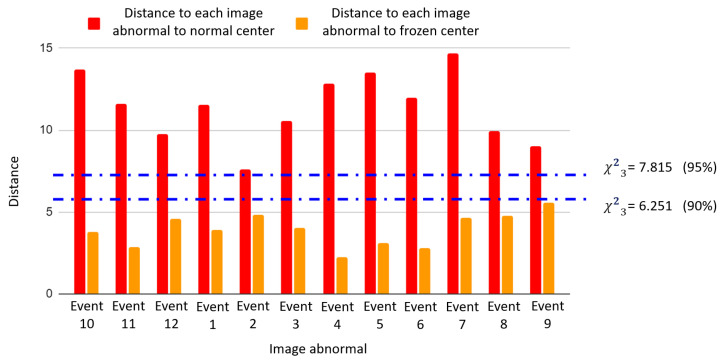
Based on the 6-feature image representation, distances from projected events to the center of the healthy condition (red) and the center of the frozen sensor fault (orange).

Figure 11 shows the latent space obtained when using correlation as the sixth descriptor, while Figure 12 depicts the corresponding latent-space-based HIs.

The comparison of Figure 9 and Figure 11 suggests that including the correlation as a feature impacts the latent space distribution, with an increase in cluster dispersion. The faulty clusters are well disentangled in the five-feature and six-feature input settings, even though some clusters remain superposed with the normal-condition cluster (e.g., spark).

Regarding the reconstruction-error-based HI, the comparison of Figure 10 and Figure 12 suggests that for the electromechanical system under investigation, the five-feature model performs better. This result means that the five-feature model captures enough information to differentiate between normal and faulty sensor conditions. The six-feature model, while still functional, causes image distortion due to the increased correlation between pixels. The comparison between the five-feature and the six-feature images highlights the importance of carefully selecting the features used in the model. Further investigation is needed to determine the pertinence of including other features to enhance the detection capabilities and adaptability to various operational scenarios.

Further evaluation is required to better assess the model’s performance in detecting sensor failures, including estimating the performance with data from different types of failure. False positives and false negatives can occur due to the inherent variability in sensor signals and the nature of the CVAE-based failure detection approach.

### 4.5. Comparison with Competing Approaches

This subsection compares the proposed CVAE-based sensor failure detection approach with existing methods in the literature, considering both the scope of detectable sensor failures and methodological aspects. Table 1 lists existing sensor failure detection methods from diverse application domains, along with the proposed approach.

The literature summarized in Table 1 highlights that previous studies predominantly focused on specific subsets of sensor failure modes. For instance, approaches presented in [15,17,20,22,24,28,47] detected isolated failure types among bias, noise, saturation, or absence of signal. In contrast, the proposed CVAE-based approach simultaneously detects the multiple listed sensor failures. Some of the listed works also addressed multiple types of sensor failure, notably [7,16,19,23].

None of the works listed in Table 1 use the CVAE model exploited in the present paper. Instead, these studies employed ML or statistical methods tailored to their respective application domains, such as classification models, probabilistic frameworks, or hybrid approaches. While these methods demonstrated effectiveness in their specific contexts, they often relied on predefined failure patterns, making them less adaptable to complex and diverse failure scenarios. The CVAE approach, in contrast, leverages a learned latent representation to generalize across different failure types without requiring explicit modeling of specific failure modes. This ability to learn a structured latent space is a significant advantage when handling unseen or ambiguous sensor faults.

The clear clustering in the CVAE latent space (as depicted in Figure 9 and Figure 11) and the clear discrimination through reconstruction error distributions (as depicted in Figure 10 and Figure 12) suggest robust detection capabilities of the proposed approach. Yet, this paper analysis highlights that including extra features does not necessarily yield performance improvement, underscoring the critical role of domain-informed feature selection. This aspect of AI modeling has also been mentioned in previous works [48].

However, it is important to highlight the difficulty of direct quantitative comparison with the competing works. Firstly, significant differences exist in the datasets used across studies, spanning various operational domains such as electrical systems, aeronautics, energy conversion systems, and health monitoring. These domain-specific datasets differ in complexity, number of sensors, sampling rates, and available types of labeled failures. The dataset used in this study, specifically from the aeronautics domain and supplemented with synthetic data, presents a unique operational context that limits comparability. Secondly, methodological and metric variability further complicate direct benchmarking. While the findings suggest promising detection performance within this specific context, further investigation is needed to generalize to other application domains and data configurations.

In summary, the primary contribution of this work is in demonstrating the feasibility and effectiveness of simultaneously detecting multiple sensor failures using a CVAE-based method within aeronautical systems. However, recognizing the inherent comparability limitations, future research should focus on validating and extending these findings across diverse datasets and standardized testing frameworks.

## 5. Conclusions

This work introduced an approach suitable for detecting multiple sensor failures in real-life systems. The proposed approach combines an image representation of statistical features with the CVAE model. While the image representation of statistical features illustrates the challenges of directly analyzing system conditions from sensor data, the CVAE encoding allows for compressing the image representation into a low-dimensional latent space.

The clear discrimination between normal and abnormal events in the CVAE latent space attests to the potential of the proposed approach. The proposed detection combined two individual HIs, one reconstruction-error-based HI and one CVAE-latent-space-based HI. This dual-metric detection was designed to enhance the model’s sensitivity to multiple sensor failures, making it suitable for critical real-life systems, such as systems from the aeronautical industry. The validation with a dataset from an aeronautical system demonstrated the ability of the proposed approach to detect sensor failures. The statistical test using the HI yields satisfactory results, with the proven capability of identifying multiple failure conditions. Moreover, it was shown that the CVAE latent space provides a convenient visualization tool with disentangled clusters representing distinct sensor failures.

The comparison between the results for five- and six-feature image representations highlighted the need to perform feature selection considering both the system characteristics and the performance of the approach under consideration. Further investigation directions include evaluating the detection approach’s performance on a broader test dataset and evaluating the proposed approach’s performance against competing detection approaches.

## Figures and Tables

**Table 1:** Summary of approaches for detecting sensor failures.

Reference	No Signal	Saturation	Bias	Frozen	Noise	Spark	Domain	Method
Our proposition	x	x	x	x	x	x	Aeronautics	ML (CVAE)
[7]	x		x		x		Electrical	ML
[15]			x				Aeronautics	Statistics
[20]					x		Energy	ML
[16]			x	x		x	Electrical	ML
[17]	x						Manufacture	ML-Statistics
[26]				x		x	Health surveillance	ML-Statistics
[28]			x				Industry 4.0	ML
[19]			x	x		x	CPS	ML
[21]	x				x		Aeronautics	ML
[23]			x	x	x	x	Aeronautics	ML
[22]			x				Energy	ML
[24]		x					Health monitoring	Statistics
[47]			x				IoT	ML

“x” identifies methods addressing the indicated issue. IoT: Internet of Things, CPS: Cyber–Physical Systems.

## Data Availability

The datasets presented in this article are not readily available because they were provided under confidentiality agreements restricting their public disclosure.
